# Survey Questions on Quantity and Frequency Are Differentially Effective by Age in Predicting Future Alcohol Consumption

**DOI:** 10.1111/dar.70019

**Published:** 2025-08-18

**Authors:** Sarah Callinan, Simon D'Aquino, Ben Riordan, Jonas Raninen, Michael Livingston, Paul M. Dietze, Gerhard Gmel, Robin Room

**Affiliations:** ^1^ Centre for Alcohol Policy Research La Trobe University Melbourne Australia; ^2^ Department of Clinical Neuroscience Karolinska Institutet Stockholm Sweden; ^3^ National Drug Research Institute Curtin University Melbourne Australia; ^4^ Behaviours and Health Risks Program Burnet Institute Melbourne Australia; ^5^ Addiction Medicine Lausanne University Hospital and University of Lausanne Lausanne Switzerland; ^6^ Research Department Addiction Switzerland Lausanne Switzerland; ^7^ Centre for Addiction and Mental Health Institute for Mental Health Policy Research Toronto Canada; ^8^ Centre for Social Research on Alcohol and Drugs Stockholm University Stockholm Sweden

**Keywords:** alcohol, AUDIT, longitudinal

## Abstract

**Introduction:**

Cross sectional research has demonstrated that screening tool questions on frequency of alcohol consumption are a better predictor of dependence and harmful drinking in younger adults; questions about quantity per occasion are a better predictor in older adults. The aim of this study is to see if this relationship also holds longitudinally.

**Methods:**

A total of 9076 respondents aged 15 and over completed at least two waves of the longitudinal annual Household Income and Labour Dynamics in Australia survey 10 years apart between 2001–2010 and 2012–2020. Standardised scores from responses to questions on drinking quantity and frequency in the first survey were used to predict consumption 10 years later in groups stratified by age.

**Results:**

Frequency of consumption was a significantly better predictor of future consumption than quantity in younger drinkers (aged < 36; *β* = 9.3, 95% confidence interval [CI] 8.6–10.0), than older drinkers (aged > 49; *β* = 5.1, 95% CI 4.8–5.5) while quantity was a better predictor in older drinkers (*β* = 8.2, 95% CI 7.2–9.3) than younger drinkers (*β* = 3.4, 95% CI 3.1–3.7).

**Discussion and Conclusions:**

Some commonly used screening items, such as drinking quantity and frequency, are differentially effective at identifying future heavy drinkers between age groups. Development of age‐specific screening tools could potentially lead to more accurate identification of people who could benefit from intervention to reduce their alcohol consumption.

## 1 Introduction

Alcohol has a causal role in 3.8% of global deaths and 4.6% of global disability [1], with over 200 health conditions associated with alcohol consumption including liver disease, cancer, and suicide [2]. Half of these harms are experienced by the relatively small number of those who drink heavily or have an alcohol use disorder (AUD) [3]. In order to offer effective interventions and limit harms, it is important to identify those who are currently drinking heavily and, importantly, those who are likely to continue to drink heavily. One of the complications in doing this is that drinking patterns change with age; on average, young people drink less often but drink more when they do, and as they age, they start to drink more often but drink less per occasion [4, 5]. This shift in how often people drink (frequency) and how much they drink per occasion (quantity) as they age matters because researchers and clinicians use questions assessing quantity and frequency when screening for alcohol‐related problems.

A common first step in AUD diagnosis is first getting a sufficiently high score in a screening tool to warrant clinical assessment. The Alcohol Use Disorders Identification Test (AUDIT) is the most widely used screening tool of harmful drinking, translated into 51 languages [6]. Most of the score from the 10‐item AUDIT comes from the three AUDIT‐C questions [7], which ask about: (i) how often they consume alcohol (frequency); (ii) how much alcohol is consumed on a usual occasion (quantity); and (iii) how often respondents drink at a risky level. Cross‐sectional work from Australia [8] has demonstrated that the item on quantity per occasion is a much weaker predictor of scores on dependence or harm in younger drinkers than in older drinkers. Furthermore, the question on frequency had an increasingly weak link with harmful drinking or dependence with increasing age—including no significant relationship at all with dependence in people aged 73 and over. Thus, frequency may be a poor predictor of harmful consumption among older people, while quantity may be a poorer predictor of harmful consumption in younger people.

The longitudinal predictive power of quantity and frequency of alcohol consumption, commonly used in screening tools, is important. Most cases of AUD are not identified [9], and the average time between onset and diagnosis is thought to be between 8 and 14 years [10, 11]. Furthermore, there are indications that assessment tools for AUD over‐identify young drinkers, a portion of whom will reduce their consumption with age without any intervention, a phenomenon referred to as teen‐transient false positives [12]. There is evidence to suggest that this is linked to the prevalence of high quantity, low frequency drinking among younger drinkers [12]. Therefore, it may be that drinkers who participate in heavy episodic drinking while they are young, without other signs of risky use, are less likely to need intervention to reduce their consumption in the long term, while frequent consumption may be a stronger predictor of future harmful drinking. Conversely, frequent consumption in older drinkers may not be a strong predictor of harmful drinking, particularly when compared to higher quantity consumed per occasion. More accurate identification of people whose heavy drinking will continue (without intervention) could lead to better allocation of resources.

While cross‐sectional data has been used to identify these patterns, there has been no work done on how these commonly assessed constructs of quantity and frequency differentially predict future consumption, longitudinally. This is an important gap as adjusting current screening tools to more accurately identify those who will continue to experience alcohol‐related problems could help to appropriately allocate resources. The aim of this study is to see how well measures of drinking quantity and frequency predict drinking behaviour 10 years later among different age groups. Given the importance of these constructs in alcohol research, the focus here will be on how well a measure of quantity and frequency can predict long‐term alcohol consumption. Based on cross‐sectional findings [8] we hypothesise that:

1. In younger drinkers, the question on frequency of consumption at T1 will be a stronger predictor than quantity at T1 of average drinks per day at T2.
2. In older drinkers, the question on quantity of consumption at T1 will be a stronger predictor than frequency at T1 of average drinks per day at T2.

## 2 Methods

### 2.1 Design

Measures of two constructs commonly assessed in screening tools such as the AUDIT, quantity and frequency of consumption, 10 years apart, were generated from a longitudinal survey conducted annually from 2001. Any respondents in the Household Income and Labour Dynamics in Australia (HILDA) survey (see below) who completed two surveys with identical alcohol questions 10 years apart were included. As the alcohol survey questions changed between Wave 1 and Wave 2, all analyses start at Wave 2—as such, Wave 11 data was also not used as it would only be used to match with Wave 1. As such, data for Time 1 was taken from Waves 2 (2002) to Wave 10 (2010) and data for Time 2 was taken from Wave 12 (2012) to Wave 20 (2020). Analyses from this paper were not pre‐registered. Responses to questions on alcohol consumption in Time 1 that each respondent participated in are used as predictors of alcohol consumption in each participant's Time 2.

### 2.2 Sample and Survey

The HILDA study is a longitudinal household‐based panel study that started in 2001 with annual surveys administered to all responding participants. While the focus of the study is on income and labour, the survey is comprehensive, including questions on alcohol consumption. All data are taken from the first twenty waves of the HILDA study [13].

For the HILDA study, households were selected in a multi‐stage process in order to form a large national probability sample of Australian households occupying private dwellings. The first wave was administered to 13,969 individuals aged 15 and over living in 7682 households. All members of the household are followed, and if they move house, they are contacted at their new address, and the new members of the household are recruited to the study. The initial household response rate was 59%, with 92% of people aged 15+ within selected households completing the initial survey. Retention rates in the next 19 waves ranged from 87% to 97%. A top‐up sample was added to the study in 2011 to increase the representativeness of the survey of the general Australian population.

Each year, multiple surveys are completed in participating households using primarily face‐to‐face interviews. In addition, there is a self‐administered paper questionnaire which is completed by each household member aged 15 or over, in their own time, after the initial interview. These questionnaires, which include the alcohol‐related questions, are then collected later. Completion of this survey from those who participated in the interviews ranged from 87.6% to 93.5%. Response and retention rates for the survey overall and the self‐completed survey are shown in Table S1.

In Waves 2 (2002) to Wave 10 (2010) and Wave 12 (2012) to Wave 20 (2020) there were 33,347 people who completed at least one survey. Of these participants, 17,296 were eligible (on the grounds of the whether or not they lived in a household that was part of the study) to complete at least one wave between 2002–2010 and 2012–2020 (required to be included in this study). Of these participants, 16,159 (93%) completed the questions on quantity and frequency of alcohol consumed at least once. Of these participants, 11,347 (70%) completed these questions in any two waves exactly 10 years apart. There were some systematic differences between those respondents who met this criterion and those who did not. As can be seen in Table S2, men and younger respondents were less likely to have two waves of alcohol consumption data 10 years apart. While there were also some differences in alcohol consumption, the demographic variables made up 18.9% of the variance compared to the 19.4% of the variance accounted for in the second model with alcohol variables included. Of these, a further 2271 (20%) were dropped from analyses as they reported no alcohol consumption at Time 1. As the area of interest was in how different drinking patterns can predict future consumption, these respondents were excluded. In cases of individuals completing multiple pairs of surveys 10 years apart, only the first eligible 10‐year time interval is used. This was done to increase the number of respondents in the younger age group. A sensitivity analysis was run with the latest pair of waves each respondent had included instead, and this made no difference to any of the results presented below. The number of respondents who are included in the study (*N*_total_ = 9076) per wave ranged from 221 (8th and 18th wave) to 6211 (2nd and 12th wave). The mean age of participants in their first wave was 39.2 (95% confidence interval 38.9, 39.5), and 48.5% of respondents were male. The number of participants in each age group is shown in Table S3.

The HILDA study is designed and managed by the Melbourne Institute of Applied Economic and Social Research at the University of Melbourne, and the data collection was approved by the University of Melbourne Human Research Ethics Committee (ID 1647030). Ethics approval for this secondary analysis of the data was approved by La Trobe University HREC (ID HE18343).

### 2.3 Measures

Respondents were asked “*Do you drink alcohol?*” with response categories including a range of frequencies of consumption (e.g., 2–3 days a month, 3–4 days a week etc.). A follow up question for drinkers asks, “On a day that you have an alcoholic drink, how many standard drinks do you usually have?” with seven response options ranging from 1 to 2 standard drinks to 13 or more standard drinks (a standard drink in Australia is 10 g of ethanol); the response frame is the last 12 months. Frequency of drinking and quantity per drinking day was coded as the midpoint of the range provided (i.e., a respondent who stated that they drank 3–4 drinks, 1–2 times per week was designated as consuming 3.5 drinks for 78 occasions per year (1.5 days per week *52 weeks per year = 78 occasions)). Annual volume, the outcome variable, is measured in standard drinks and calculated as the product of the quantity per occasion and the number of occasions per year (e.g., 3.5 drinks per occasion *78 occasions = 273 drinks for the example above). Respondents who stated that they had not consumed alcohol in a given year were designated as 0 on the measure of total volume.

Respondents were also grouped into categories based on their age at Time 1 (shown in Table 1). Due to smaller sample sizes than when this analyses has been completed previously using cross‐sectional data [8]—only four age groups were used. As respondents were included in the study from age 15 and the legal drinking age in Australia is 18, analyses were also run with the 15–24 year old group split into 15–17 and 18–24 year old groups. As there was no real difference in the estimates, but increased confidence intervals from reduced power, analyses are shown with the single 15–24 year old age group.

**TABLE 1:** Mean drinks per occasion (quantity) and number of drinking occasions per year (frequency) by time and age.

T1 Age	Frequency (Drinking occasions per year) (95% CIs)		Quantity (Drinks per usual occasion) (95% CIs)		Total volume (drinks per week) (95% CIs)		*N*
	Time 1	Time 2	Time 1	Time 2	Time 1	Time 2	
15–24	56.2 (53.2, 59.3)	77.2 (73.3, 81.1)	4.7 (4.5, 4.8)	3.7 (3.5, 3.8)	5.6 (5.2, 6.1)	6.3 (5.8, 6.7)	1982
25–36	91.9 (87.9, 96.0)	98.7 (94.2, 103.3)	3.6 (3.5, 3.7)	3.0 (2.9, 3.1)	7.0 (5.6, 7.5)	6.9 (6.4, 7.4)	2237
37–49	125.7 (120.7, 130.8)	127.7 (122.5, 132.8)	3.0 (2.9, 3.1)	2.7 (2.6, 2.8)	8.1 (7.6, 8.5)	8.3 (7.8, 8.8)	2436
50+	148.1 (142.2, 154.0)	135.2 (129.3, 141.0)	2.5 (2.5, 2.6)	2.1 (2.0, 2.1)	8.6 (8.1, 9.1)	7.3 (6.8, 7.7)	2421
Total	108.2 (105.3, 111.1)	111.5 (108.9, 114.1)	3.4 (3.3, 3.4)	2.8 (2.7, 2.9)	7.4 (7.2, 7.7)	7.2 (7.0, 7.5)	9076

Abbreviation: CI, confidence interval.

### 2.4 Analysis

Fixed effects regression models predicting total volume consumed at Time 2, with standardised frequency and quantity questions at Time 1 as predictors, with quantity × age and frequency × age interactions generated to assess the strength of quantity and frequency as predictors over age groups. To enable direct comparisons of the relative strength of quantity and frequency as predictors, both variables were standardised by converting them into *z*‐scores (with a mean of 0 and a standard deviation of 1). Frequency of drinking and quantity per occasion did not violate the assumption of multicollinearity (*r* = 0.24), and non‐overlap of 95% confidence intervals was used to determine significant differences in regression coefficients. Please note that as the aim of the study was to assess how quantity and frequency can predict future consumption, no covariates were added to the models as these factors would not be accounted for by a screening tool. A sensitivity analysis, to ensure that the fairly arbitrary selection of a 10‐year gap between Time 1 and 2 was not unduly affecting results, was conducted with 5 (*N* = 11,476) or 15 (*N* = 7040) year intervals used instead of 10. The process of selecting respondents for the dataset with a 10‐year gap was duplicated to generate both of these datasets. As some respondents were clustered within households, all standard errors in both the descriptive statistics and regression models were corrected for this through the *cluster* specification in the xtreg command.

## 3 Results

The drinking patterns of each age group at both time points are shown in Table 1. Please note that for these analyses Time 1 is the first wave that is included for each individual in the survey (between 2002 and 2011) and Time 2 (between 2012 and 2021) is 10 years later for each respondent. Frequency of consumption rose with age at both time points except between 37–49 and 50+ in Time 2 and increased with time in drinkers aged 15–24. Quantity per occasion decreased with age and also with time in all age groups.

**FIGURE 1:**
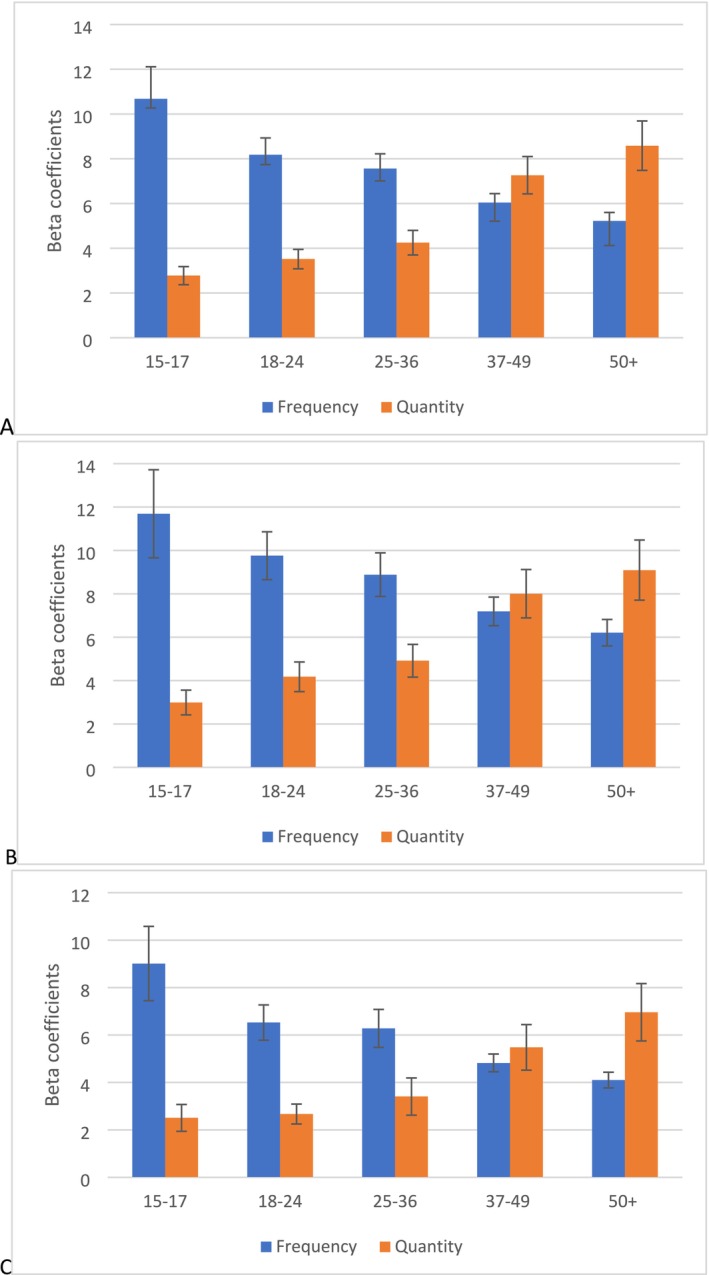
Regression coefficients for frequency and quantity predicting total volume 10‐years later for each Time 1 age group in the full sample (A), in men (B) and women (C). *N* = 9076.

Fixed effects regression models were run, predicting total volume of consumption for each participant in their Time 2, using standardised quantity and frequency of consumption measures in their Time 1 as predictors. Coefficients from these models are shown in Figure 1 and can also be found, with confidence interval values, in Table S4. As can be seen in Figure 1A, quantity and frequency at Time 1 were both positive, significant predictors of total volume 10 years later. However, using 95% confidence intervals to compare the coefficients of standardised variables, frequency of consumption was a better predictor in younger age groups (under 36 years old) and quantity per occasion was a better predictor in those aged 50 and over. The same was true in men and in women (as defined by respondents, shown in Figure 1B,C) albeit without wider confidence intervals and less significant differences.

**FIGURE 2:**
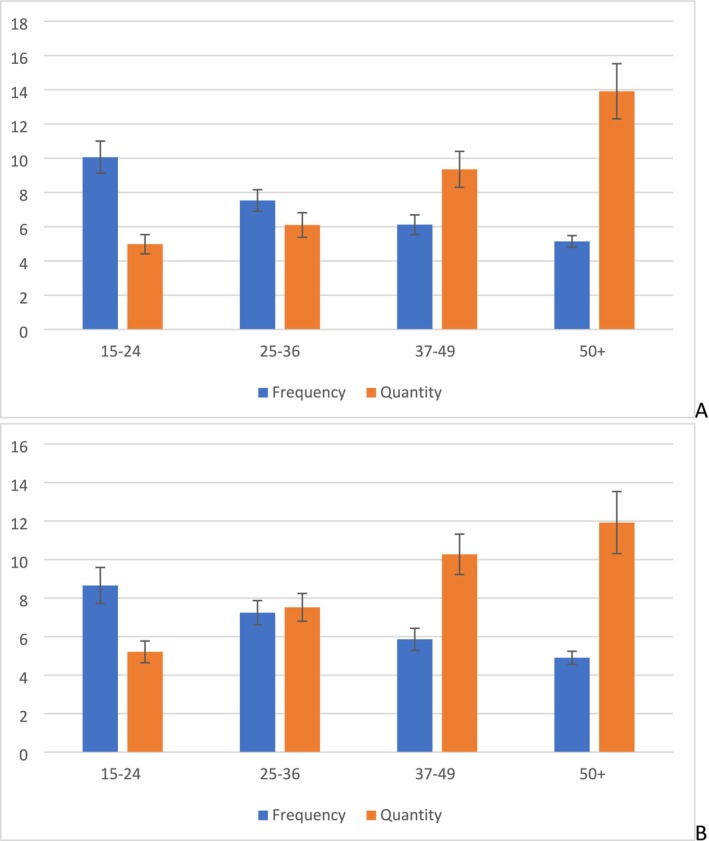
Regression coefficients for frequency and quantity predicting total volume five (A) and 15 (B) years later for each Time 1 age group. *N* = 11,476 (A) and 7040 (B).

This finding is remarkably robust: the regression coefficients when the length of the interval between Time 1 and Time 2 is five (A) or 15 (B) years are shown in Figure 2. These figures are very similar to the ones shown in Figure 1—regardless of the interval length, frequency is a better predictor of future consumption in young people, and quantity is a better predictor for older drinkers.

## 4 Discussion

We used longitudinal data to assess the efficacy of regularly used survey items on quantity and frequency of alcohol consumption to predict future total consumption. As predicted, the question on frequency of consumption was a significantly stronger predictor of future total consumption than the item on quantity per occasion in younger drinkers (those aged under 25). Furthermore, the item on quantity per occasion was a better predictor of future consumption in older drinkers (older than 49). The age at which this crossover occurs is less clear; there was no significant difference between quantity and frequency as a predictor for men and women aged 37–49 or for respondents aged 25–36 in the sensitivity analysis with a 15‐year interval.

This study aligns with cross sectional research that also found that questions on quantity and frequency are differentially predictive of concurrent dependence and harmful drinking [8]. However, unlike previous research, this study found that baseline frequency still remained a useful predictor of future consumption in older drinkers. Nevertheless, it should be noted that in this paper the outcome variable is future consumption, rather than a measure of harmful drinking or dependence. This study provides further evidence that these two dimensions of consumption are differentially predictive of future consumption, adding to previous work that finds it predictive of cross sectional harmful drinking [8].

Our findings speak to the importance of accounting for differing drinking patterns between demographic groups to increase the accuracy of screening tools that aim to identify people who could benefit from intervention or treatment. That said, it is also important to note that we have not used these questions to assess how they predict an actual future diagnosi; this is an important topic for future research. Importantly, screening tools aren't just used to predict dependence; they are also used to predict those who do not meet this criterion but could still benefit from intervention. So, this work still has important implications for how we identify people who may continue to drink heavily.

One of the current issues with screening tools is that they tend to over‐identify young people, most notably teen transient drinkers, those who meet AUD criteria when they are younger but go on to reduce consumption without intervention as they age [12]. Ideally, a screening tool could more specifically identify those who will not reduce their consumption with age. This could in turn lead to a focus on those who are more likely to continue to drink heavily and directing fewer resources toward those who are more likely to reduce their consumption without intervention. Survey items focused on frequency of drinking could be the key to this. As noted above, young people tend to drink more per occasion, less often. This drinking pattern might be the one that is more closely linked with teen transient drinking, while drinking more frequently when young might be a better indicator that a drinker is less likely to decrease their consumption without intervention. This aligns with previous work that found that some of the alcohol use disorder diagnostic criteria linked with heavy episodic drinking are those most strongly linked with teen transient drinking [12]. Therefore, these items on heavy episodic drinking may be useful predictors of future consumption; however, in younger people, the predictive value of heavy episodic drinking may be confounded by a large portion of younger drinkers participating in heavy episodic drinking more temporarily in their youth. Furthermore, as frequency is not as good a predictor of future consumption in older drinkers, it appears that these questions could be differentially effective by age. Overall, identification of younger drinkers who drink like an older drinker (frequently) and older drinkers who drink like a younger drinker (more per occasion) could lead to better allocation of resources. A shift in the scoring of these items, or even possibly exclusion of poorly performing items in specific age groups, could lead to more accurate identification of those who would best benefit from intervention.

The primary limitation of this study is that inherent to all survey research—people's responses on these surveys are subject to a number of biases, including social desirability [14]. Furthermore, while the response and retention rates of the HILDA dataset are admirable by Australian standards [15], there is still the potential for systematic under‐ or over‐sampling of given populations in our sample. Furthermore, within the sample recruited, there were differences, primarily demographic, found between those who had completed one wave of the survey but not met the criterion of having two waves of the survey 10 years apart and those who did meet said criterion. The analyses were not pre‐registered; so the results should be considered exploratory. Finally, we have no information on people who accessed treatment during the time between the two surveys—while we would expect that the vast majority of the sample would not have accessed treatment in this time, there will presumably be a small number who would have done so—this could have impacted on our results.

In conclusion, young people who drink frequently drink more alcohol 10 years later than those who drink more per occasion, while older drinkers who drink more per occasion will drink more alcohol 10 years later than those who drink more frequently. Given that young people drink more per occasion and older drinkers drink more frequently, this means that particular attention should be paid to older drinkers who drink like they are young (heavily per occasion) and younger drinkers who drink like they are old (more frequently). This information can be used to develop more accurate and targeted screening tools to identify those that could most benefit from treatment or intervention.

## Author Contributions

Conceptualisation: S.C. (Lead); Formal Analysis S.C. (Equal) S.D. (Equal); Methodology S.C. (Lead), Writing – original draft S.C. (Lead) S.D., B.R., J.R., M.L., P.M.D., G.G., R.R. (Supporting). Writing – review and editing S.C., S.D., B.R., J.R., M.L., P.M.D., G.G., R.R. (Equal).

## Conflicts of Interest

The authors declare no conflicts of interest.

## Supporting information


**Data S1:** Supporting Information.

## Data Availability

Data sharing is not applicable to this article as no new data were created or analyzed in this study.
