# Supplementary material for: Survey Questions on Quantity and Frequency Are Differentially Effective by Age in Predicting Future Alcohol Consumption
Source: Drug Alcohol Rev. 2025 Aug 18;44(7):1961–7. doi: 10.1111/dar.70019 (PMC12581926; doi:10.1111/dar.70019)
Supplement: Supplementary file 1 — Data S1: Supporting Information. [file DAR-44-1961-s001.docx]

Table S1. Details of Household Income and Labour Dynamics in Australia survey data used in this study

|  |  | Original sample | | Top up sample | | Total | |
| --- | --- | --- | --- | --- | --- | --- | --- |
| Wave | N | Retention | SCQ | Retention | SCQ | Retention | SCQ |
| 2001 | 13,969 | 58.8# | 93.5 |  |  |  |  |
| 2002 | 13,041 | 86.9 | 93.0 |  |  |  |  |
| 2003 | 12,728 | 90.4 | 92.3 |  |  |  |  |
| 2004 | 12,408 | 91.6 | 91.9 |  |  |  |  |
| 2005 | 12,759 | 94.4 | 89.9 |  |  |  |  |
| 2006 | 12,905 | 94.9 | 90.8 |  |  |  |  |
| 2007 | 12,789 | 94.7 | 89.0 |  |  |  |  |
| 2008 | 12,785 | 95.2 | 87.6 |  |  |  |  |
| 2009 | 13,301 | 96.3 | 86.9 |  |  |  |  |
| 2010 | 13,526 | 96.4 | 89.1 |  |  |  |  |
| 2011 | 13,603 | 96.5 | 87.8 | 63.0# | 85.2 |  | 87.2 |
| 2012 | 13,536 | 96.2 | 88.7 | 92.3 | 85.9 | 95.3 | 88.1 |
| 2013 | 13,609 | 96.4 | 88.1 | 92.7 | 86.6 | 95.5 | 87.8 |
| 2014 | 13,633 | 96.5 | 89.2 | 94.5 | 88.5 | 96.1 | 89.1 |
| 2015 | 13,753 | 97.0 | 88.5 | 95.0 | 86.8 | 96.5 | 88.1 |
| 2016 | 13,834 | 97.0 | 92.1 | 96.0 | 91.0 | 96.7 | 91.9 |
| 2017 | 13,791 | 96.7 | 92.2 | 95.3 | 90.8 | 96.4 | 91.9 |
| 2018 | 13,723 | 96.4 | 91.1 | 95.3 | 91.1 | 96.2 | 91.1 |
| 2019 | 13,748 | 96.9 | 92.2 | 96.2 | 91.7 | 96.7 | 92.1 |
| 2020 | 13,467 | 95.5 | 91.9 | 94.1 | 91.6 | 95.2 | 91.9 |

SCQ: Self Completion Questionnaire which is completed by the respondent after the initial interview. # This was the response rate, rather than the retention rate, for the first wave of each sample.

Table S2. Regression models predicting the odds of participants in one wave not participating in another 10 years later.

|  |  | Model 1 | Model 2 |
| --- | --- | --- | --- |
| Age | 1 | 1(Ref) | 1(Ref) |
|  | 2 | **0.65 (0.58, 0.74)** | 0.69 (0.58, 0.82) |
|  | 3 | **0.41 (0.36, 0.46)** | 0.44 (0.36, 0.53) |
|  | 4 | **0.37 (0.33, 0.43)** | 0.43 (0.35, 0.52) |
| Sex | Male | 1(Ref) | 1(Ref) |
|  | Female | **0.64 (0.57, 0.71)** | 0.63 (0.54, 0.74) |
| Age x sex | 2*Female | 1.13 (0.94, 1.34) | 1.14 (0.89, 1.47) |
|  | 3*Female | **1.36 (1.29, 1.64)** | 1.35 (1.03, 1.77) |
|  | 4*Female | **1.93 (1.62, 2.31)** | 1.80 (1.39, 2.33) |
| Drinking frequency | |  | **0.93 (0.90, 0.95)** |
| Quantity per occasion | |  | 1.02 (1.00, 1.04) |
| Pseudo R2 | | 0.0189 | 0.0194 |

Table S3. Participants in each wave and age group

| Wave | 15-24 year-olds^a^ | 25-36 year-olds^a^ | 37-49 year-olds^a^ | 50+ year-old^a^ | Total^b^ |
| --- | --- | --- | --- | --- | --- |
| 2 & 12 | 808 | 1,438 | 1,954 | 2,011 | 6,211 |
|  | 40.8% | 64.3% | 80.2% | 83.1% | 68.4% |
|  |  |  |  |  |  |
| 3 & 13 | 232 | 212 | 181 | 168 | 793 |
|  | 11.7% | 9.5% | 7.4% | 6.9% | 8.7% |
|  |  |  |  |  |  |
| 4 & 14 | 151 | 125 | 59 | 55 | 390 |
|  | 7.6% | 5.6% | 2.4% | 2.3% | 4.3% |
|  |  |  |  |  |  |
| 5 & 15 | 146 | 81 | 52 | 43 | 322 |
|  | 7.4% | 3.6% | 2.1% | 1.8% | 3.6% |
|  |  |  |  |  |  |
| 6 & 16 | 174 | 78 | 49 | 36 | 337 |
|  | 8.9% | 3.5% | 2.0% | 1.5% | 3.7% |
|  |  |  |  |  |  |
| 7 & 17 | 140 | 85 | 34 | 26 | 285 |
|  | 7.1% | 3.8% | 1.4% | 1.1% | 3.1% |
|  |  |  |  |  |  |
| 8 & 18 | 102 | 64 | 31 | 24 | 221 |
|  | 5.2% | 2.9% | 1.3% | 1.0% | 2.4% |
|  |  |  |  |  |  |
| 9 & 19 | 111 | 69 | 39 | 34 | 253 |
|  | 5.6% | 3.1% | 1.6% | 1.4% | 2.8% |
|  |  |  |  |  |  |
| 10 & 20 | 118 | 85 | 37 | 24 | 264 |
|  | 5.6% | 3.8% | 1.5% | 1.0% | 2.9% |
|  |  |  |  |  |  |
| Total | 1,982 | 2,237 | 2,436 | 2,421 | 9,076 |
|  | 21.8% | 24.7% | 26.8% | 26.7% | 100.0% |

^a^ Percentage by row, ^b^ percentage by column.

Table S4. Regression coefficients for frequency and quantity predicting total volume 10-years later for each age group in the full sample (A), in men (B), and women (C); and 5 and 10 years later for each age group.

| Ten-year gap | | |
| --- | --- | --- |
| Full sample | | |
|  | Frequency | Quantity |
| 15-24 | 9.30 (8.60, 10.0) | 3.39 (3.07, 3.72) |
| 25-36 | 7.57 (7.00, 8.14) | 4.25 (3.75, 4.75) |
| 37-49 | 6.18 (5.78, 6.59) | 6.88 (6.11, 7.64) |
| 50+ | 5.12 (4.76, 5.48) | 8.24 (7.15, 9.34) |
| Men | | |
|  | Frequency | Quantity |
| 15-24 | 10.34 (9.39, 11.29) | 3.90 (3.40, 4.39) |
| 25-36 | 8.73 (7.91, 9.55) | 4.78 (4.12, 5.44) |
| 37-49 | 7.33 (6.69, 7.97) | 7.35 (6.34, 8.36) |
| 50+ | 6.04 (5.47, 6.61) | 8.69 (7.30, 10.08) |
| Women | | |
|  | Frequency | Quantity |
| 15-24 | 7.78 (6.89, 8.66) | 2.71 (2.36, 3.07) |
| 25-36 | 6.34 (5.61, 7.07) | 3.56 (2.79, 4.33) |
| 37-49 | 4.99 (4.58, 5.40) | 5.76 (4.78, 6.74) |
| 50+ | 4.05 (3.72, 4.37) | 6.91 (5.55, 8.28) |
| Five-year gap | | |
|  | Frequency | Quantity |
| 15-24 | 10.06 (9.12, 10.99) | 4.98 (4.41, 5.54) |
| 25-36 | 7.53 (6.90, 8.16) | 6.10 (5.38, 6.82) |
| 37-49 | 6.12 (5.55, 6.70) | 9.35 (8.3, 10.40) |
| 50+ | 5.14 (4.80, 5.48) | 13.91 (12.30, 15.52) |
| 15-year gap | | |
|  | Frequency | Quantity |
| 15-24 | 8.65 (7.89, 9.41) | 5.21 (4.67, 5.76) |
| 25-36 | 7.24 (6.67, 7.81) | 7.52 (6.54, 8.51) |
| 37-49 | 5.86 (5.51, 6.22) | 10.27 (9.03, 11.52) |
| 50+ | 4.90 (4.50, 5.30) | 11.92 (10.3, 13.54) |
